# Complement C3 and C5 Deficiency Affects Fracture Healing

**DOI:** 10.1371/journal.pone.0081341

**Published:** 2013-11-18

**Authors:** Christian Ehrnthaller, Markus Huber-Lang, Per Nilsson, Ronny Bindl, Simon Redeker, Stefan Recknagel, Anna Rapp, Tom Mollnes, Michael Amling, Florian Gebhard, Anita Ignatius

**Affiliations:** 1 Department of Traumatology, Hand-, Plastic-, and Reconstructive Surgery, Center of Surgery, University of Ulm, Ulm, Germany; 2 Institute of Orthopedic Research and Biomechanics, Center of Musculoskeletal Research, University of Ulm, Ulm, Germany; 3 Department of Osteology and Biomechanics, University Medical Center Hamburg-Eppendorf, Hamburg, Germany; 4 Department of Immunology, Institute of Clinical Medicine, University of Oslo, Oslo, Norway; Harvard Medical School, United States of America

## Abstract

There is increasing evidence that complement may play a role in bone development. Our previous studies demonstrated that the key complement receptor C5aR was strongly expressed in the fracture callus not only by immune cells but also by bone cells and chondroblasts, indicating a function in bone repair. To further elucidate the role of complement in bone healing, this study investigated fracture healing in mice in the absence of the key complement molecules C3 and C5. C3^-/-^ and C5^-/-^ as well as the corresponding wildtype mice received a standardized femur osteotomy, which was stabilized using an external fixator. Fracture healing was investigated after 7 and 21 days using histological, micro-computed tomography and biomechanical measurements. In the early phase of fracture healing, reduced callus area (C3^-/-^: -25%, p=0.02; C5^-/-^: -20% p=0.052) and newly formed bone (C3^-/-^: -38%, p=0.01; C5^-/-^: -52%, p=0.009) was found in both C3- and C5-deficient mice. After 21 days, healing was successful in the absence of C3, whereas in C5-deficient mice fracture repair was significantly reduced, which was confirmed by a reduced bending stiffness (-45%; p=0.029) and a smaller callus volume (-17%; p=0.039). We further demonstrated that C5a was activated in C3^-/-^ mice, suggesting cleavage via extrinsic pathways. Our results suggest that the activation of the terminal complement cascade in particular may be crucial for successful fracture healing.

## Introduction

It is known that the immune and skeletal systems are closely linked [1], because both share crucial regulatory cytokines, the most prominent being the receptor activator of nuclear factor-κB ligand (RANKL), which is released by osteoblasts and also by immune cells, stimulating osteoclast formation and activity [2,3]. There is increasing evidence that the complement system, a crucial part of innate immunity, may also play an important role in bone biology [4,5]. The complement system is a potent trigger and amplifier of local and systemic inflammation. The complement cascade consists of almost 40 different proteases that are activated in a chain-reaction by three main activation pathways (classic, alternative and lectin pathways). All three pathways result in the generation of the anaphylatoxins C3a and C5a, which, via binding to their corresponding receptors C3aR and C5aR, induce important functions of immune cells, including cytokine release and cell migration [4]. Additionally, complement-independent pathways exist, such as cross-activation with other serine protease systems (e.g. the coagulation system) [6] or cellular activation by macrophages [7]. Extrinsic complement activation is regarded as an important mechanism in traumatic injuries [6].

The regulatory role of complement in bone biology became evident from clinical observance and a number of experimental studies. Complement proteins are involved in the pathogenesis of several diseases also affecting bone, including complement-induced systemic lupus erythematosus [8] and rheumatoid arthritis [9], both leading to bone loss. Furthermore, it was shown that complement proteins are expressed in a distinct spatial pattern in the growth plate during bone development [10]. C1s, the first component involved in classic pathway activation, was found in hypertrophic chondrocytes and in degrading matrix during endochondral ossification [11-13]. Because C1s is able to cleave collagen [14] and activate other matrix-degrading enzymes [13], it was suggested to potentially play a role in cartilage-bone transformation during endochondral ossification [15]. Furthermore, several authors demonstrated differential regulation of complement-related genes during osteogenic differentiation, indicating an important role in osteoblast development and function [16-18]. Osteoblasts are able to generate the key complement proteins C3 and C5 [19,20], and express the anaphylatoxin receptors C3aR and C5aR, which mediate osteoblast migration and the release of pro-inflammatory cytokines [17,21,22]. A recent study of our group demonstrated that osteoclasts could actively cleave C5 to functional C5a and that C5a induced osteoclastogenesis [19].

Based on these reports, we proposed that complement may also be involved in fracture healing. To investigate whether the cells in the fracture callus are target cells for activated complement, we previously analyzed the expression of the key receptor C5aR during fracture healing in rats [17]. As expected, C5aR was expressed by immune cells present in the fracture hematoma, but was also abundantly expressed by osteoblasts, chondroblasts and osteoclasts in zones of intra-membranous and endochondral ossification during all stages of fracture healing. Therefore, complement may not only play a role in the inflammatory phase but also in the repair phase of fracture healing [17]. 

In the present study we investigated fracture healing in C3- and C5-deficient mice to further elucidate the role of complement in bone regeneration. C3 and C5 are cleaved into the potent anaphylatoxins C3a and C5a, which act as key factors in the complement cascade. Because C5 can be extrinsically cross-activated by the thrombin pathway, which is regarded as an important activation mechanism in traumatic injuries [6], we hypothesized that fracture healing would not be affected in the absence of C3 but would be in the absence of C5.

## Materials and Methods

### Mouse models

All experiments followed the international regulations for the care and use of laboratory animals after the approval of the national ethical committee (Germany, Regierungspräsidium Tübingen, No. 965). All surgery was performed under general anesthesia, and all efforts were made to minimize suffering.

Mice were purchased from Jackson Laboratories (Bar Harbor, Maine, USA).

 C3-deficient mice (B6;129S4-*C3tm1Crr*/J; C3^-/-^) were used with no detectable C3 in their serum [23]. C57BL/6J mice were chosen as corresponding controls, as backcrossing for five generations was performed with this strain.

 C5-deficient mice (B10.D2-*Hc0 H2d H2*-*T18c*/oSnJ; C5^-/-^) carry the *Hc0* allele from DBA/2J, making them deficient for C5 [24]. C57BL/10SnJ mice serving as the backcrossing strain were chosen as controls.

### Bone characterization

To investigate the bone phenotype of C3^-/-^ and C5^-/-^ mice, Xrays (Faxitron Bioptics, Tucson, USA) of their skeletons were performed. Femora and lumbar vertebrae were then harvested. The femora were biomechanically analyzed using a three-point bending test. Micro-computed tomography (µCT) scanning was performed with vertebral body L6 and with femora using the Skyscan 1172 (Skyscan, Kontich, Belgium). Evaluation was performed in accordance with the recommendations of the American Society of Bone and Mineral Research (ASBMR) [25].

### Fracture healing experiments

One hundred and fifty-seven male mice, aged 12 weeks, were divided into 4 experimental groups: C3^-/-^ mice (n = 39) and their corresponding wildtype controls (n = 40) as well as C5^-/-^ mice (n = 38) and their corresponding wildtype controls (n = 40). All mice received analgesic in the drinking water from 2 days preoperatively to 3 days postoperatively (25 mg/L, Tramal^®^, Gruenenthal, Aachen, Germany). After subcutaneous injection of atropine sulfate (50 μg/kg, Atropin^®^, Braun, Melsungen, Germany), the mice were anesthetized using 2% isoflurane (Forene^®^, Abbott, Wiesbaden, Germany). Antibiotic prophylaxis with clindamycin was performed for 3 days postoperatively (45 mg/kg, Sobelin 600^®^, Pfizer Pharma, Karlsruhe, Germany). The surgical procedure was described in detail previously [26]. Briefly, a standardized osteotomy gap of 0.4 mm was created at the midshaft of the right femur and stabilized with an external fixator using 4 mini-Schanz screws (Research Implant System, RIS, Davos, Switzerland). After a healing period of 7 and 21 days, animals were sacrificed. Additional animals were sacrificed for serum analysis on days 1 and 3.

### Serum C3a and C5a measurements

Serum C3a was analyzed using commercially available C3a antibodies (558250, BD Biosciences, Franklin Lakes, New Jersey, USA) by conventional ELISA methods. Serum C5a was analyzed using a commercially available ELISA kit (DY2150) in accordance with the manufacturer´s protocol (R&D Systems, Inc., Minneapolis, USA).

### Biomechanical testing

All femora explanted on day 21 were subjected to non-destructive three-point bending tests as described previously [26]. Briefly, the proximal femora were embedded in aluminum cylinders using SelfCem (Heraeus Kulzer, Hanau, Germany). Then the embedded femora were inserted into a material-testing machine (Mod. Z010, Zwick GmbH & Co., Ulm, Germany). The bending load was applied to the top of the callus and was recorded continuously versus sample deflection. At a maximum speed of 2 mm/min the maximum load was 1.5 N. 

Because the callus was not always located at the center of the supports (l/2), the distances between the load vector and the proximal (a) and distal (b) supports were considered for calculating EI = k ^a2 b2^ (Nmm^2^) [26].

### Histological analysis

After fixation in 4% formaldehyde and dehydration with ethanol, the bones were embedded in methyl methacrylate (Merck KGaA, Darmstadt, Germany). Histological slices were harvested from longitudinal cuts through the center of the bone and surface-stained with Paragon (toluidine blue and fuchsin; both Waldeck GmbH & Co KG, Münster, Germany). The slices were examined under a light microscope (Leica DMI6000B, Leica, Heerbrugg, Switzerland) at fivefold magnification. The amount of bone, cartilage and fibrous tissue was assessed by circumscribing the corresponding areas using image analysis software (Leica MMAF 1.4.0 Imaging System, powered by MetaMorph1). 

The growth plate height was analyzed in 10 mice from each group from healing time point days 1 and 3. In every histological slide, growth plate height was determined along the distal epiphyseal growth plate at 10 locations using Leica imaging analysis software (Leica MMAF 1.4.0 Imaging System). 

### IL-6 Immunostaining

Immunostaining of IL-6 was performed on paraffin-embedded sections of the fracture callus from day 7 by the use of a polyclonal goat anti-IL-6 antibody (IL-6 (M-19); Santa Cruz Biotechnology, CA) at 1 μg/mL diluted in phosphate-buffered saline (PBS) containing 1% bovine serum albumin for overnight at 4°, followed by biotinylated donkey anti- goat antibody (Invitrogen, Carlsbad, CA) for 30 minutes. For negative controls, the primary antibody was substituted with goat immunoglobulin (IgG:I5256, Sigma-Aldrich, Taufkirchen, Germany). Endogenous peroxidase activity was stopped with 3% H_2_O_2_ and unspecific binding was blocked with 2% bovine serum albumin and 0.1% Triton X-100 in tris buffer. Biotin was detected using ZytoChem-Plus streptavidin-horse radish peroxidase technology and 3-amino-9-ethylcarbazol as the chromogen (Zytomed Systems, Berlin, Germany). Finally, counterstaining with hematoxylin was performed. Analysis was performed descriptively under a light microscope (Leica DMI6000B, Leica, Heerbrugg, Switzerland).

### Micro-computed tomography (μCT)

Femora and lumbar vertebrae (L6) were scanned using a µCT device (Skyscan 1172, Skyscan) at a resolution of 8 µm and with the settings 50 kV and 200 µA.

For the fracture healing experiments, the region of interest (ROI) was defined as the periosteal callus together with the fracture gap. For bone characterization, the ROI was the mid-diaphyseal shaft of the femur and a cylinder with a cross-section of 0.8mm and the height of 1.8mm in the lumbar spine. Using CT-analysis software (Data viewer, Skyscan) the callus was segmented and the unnecessary callus regions were discarded. Global thresholding was performed to distinguish between mineralized and non-mineralized tissue. The grey value corresponding to 25% of Xray attenuation of the cortical bone of each specimen was taken as the threshold [27]. To determine the mineral density, bone phantoms with 250 and 750 mg/kg hydroxyapatite were used for calibration and the same threshold as described above was used. Common ASBMR standard parameters were evaluated (CTAnalyser, Skyscan). In all ROIs, the total tissue volume and the bone volume fraction (BV/TV) were calculated. The maximal moment of inertia was calculated based on the tissue area on the transversal slices in the fracture gap. 

### Statistical analysis

Data were expressed as mean ± standard error (SEM). Statistical analysis was performed using the unpaired t-test (IBM SPSS Statistics 19.0, SPSS Inc., IBM, Armonk, New York, USA). Results with p≤0.05 were considered significant.

## Results

### Bone phenotype

**Figure 1:**
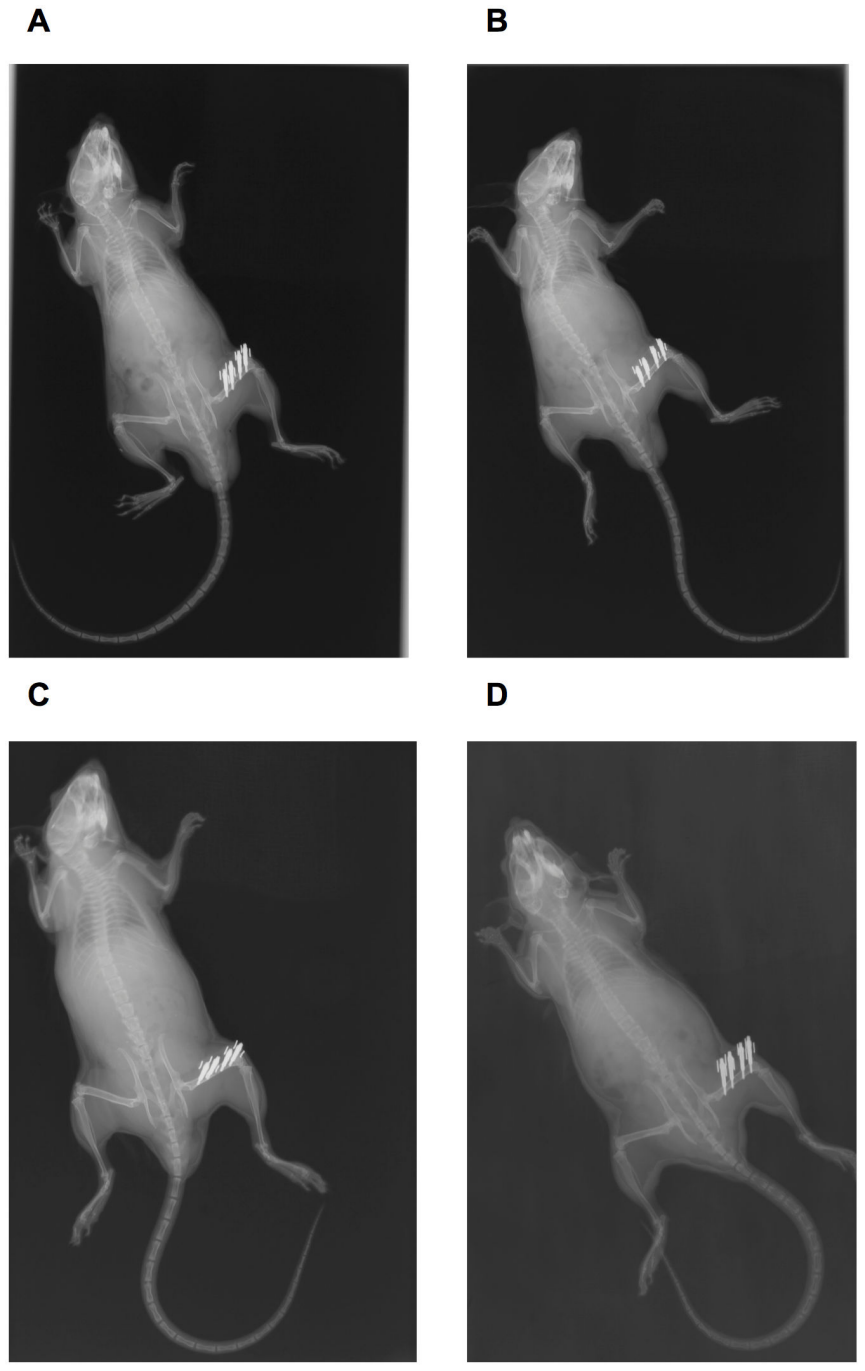
Xray examination of the skeleton. Whole-body Xrays of complement-deficient and corresponding wildtype mice immediately following surgery. The osteotomy of the right femur was stabilized using an external fixator, which was mounted with two mini-Schanz screws each in the proximal and distal bone fragment. A: C3^-/-^mouse; B: C3^+/+^ mouse; C: C5^-/-^ mouse; D: C5^+/+^ mouse.

A comparison of C3^-/-^ and C5^-/-^ mice with their corresponding wildtype controls revealed no significant macroscopic changes to their skeletons at age 12 weeks (Figure 1). Biomechanical analysis revealed a significantly increased bending stiffness of the femora of C5^+/+^ mice (+21%), corresponding to a significantly increased moment of inertia. Bending stiffness of C3^+/+^ mice was elevated (+17%) but failed to reach statistical significance.

Analysis of the trabecular bone of the lumbar spine exhibited no significant differences in BV/TV, trabecular thickness or trabecular number (Tables 1 and 2).

**Table 1:** Bone phenotype in C3^-/-^.

**Region of interest**	**Parameter**	**B6;129S4-C3^tm1Crr^/J**		**C57/Bl6J**		
		Mean	SEM	Mean	SEM	p-value
Femur diaphysis	Biomechanical stiffness (N×mm^4^)	2438.2	± 138.0	2997.8	± 118.4	0.07
cortical bone	Cortical thickness (mm)	0.22	± 0.006	0.22	± 0.004	0.40
	Moment of inertia (mm^4^)	0.32	± 0.01	0.33	± 0.01	0.76
Lumbar spine	BV/TV (%)	10.6	± 0.73	11.58	± 0.95	0.44
trabecular bone	Trabecular number (/mm)	1.75	± 0.04	1.8	± 0.14	0.47
	Trabecular thickness (mm)	0.06	± 0.003	0.06	± 0.002	0.66

Biomechanical and µCT analyses of the intact bones of C3^-/-^ mice and the corresponding wildtype mice; displayed as the mean and SEM.

**Table 2:** Bone phenotype in C5^-/-^.

**Region of interest**	**Parameter**	**B10.D2-Hc^1^ H2^d^ H2-T18^c^/nSnJ**		**C57/Bl10SnJ**		
		Mean	SEM	Mean	SEM	p-value
Femur diaphysis	Biomechanical stiffness (N×mm^4^)	3096.3	± 187.6	3930.5	± 89.1	0.01*
cortical bone	Cortical thickness (mm)	0.23	± 0.04	0.24	± 0.003	0.06
	Moment of inertia in (mm^4^)	0.37	± 0.01	0.48	± 0.03	0.02*
Lumbar spine	BV/TV (%)	13.4	± 0.8	13.9	± 0.6	0.61
trabecular bone	Trabecular number (/mm)	1.9	± 0.15	2.0	± 0.8	0.49
	Trabecular thickness (mm)	0.07	± 0.002	0.07	± 0.002	0.34

Biomechanical and µCT analyses of the intact bones of C5^-/-^ mice and the corresponding wildtype mice; displayed as the mean and SEM; *=p≤0.05

**Figure 2:**
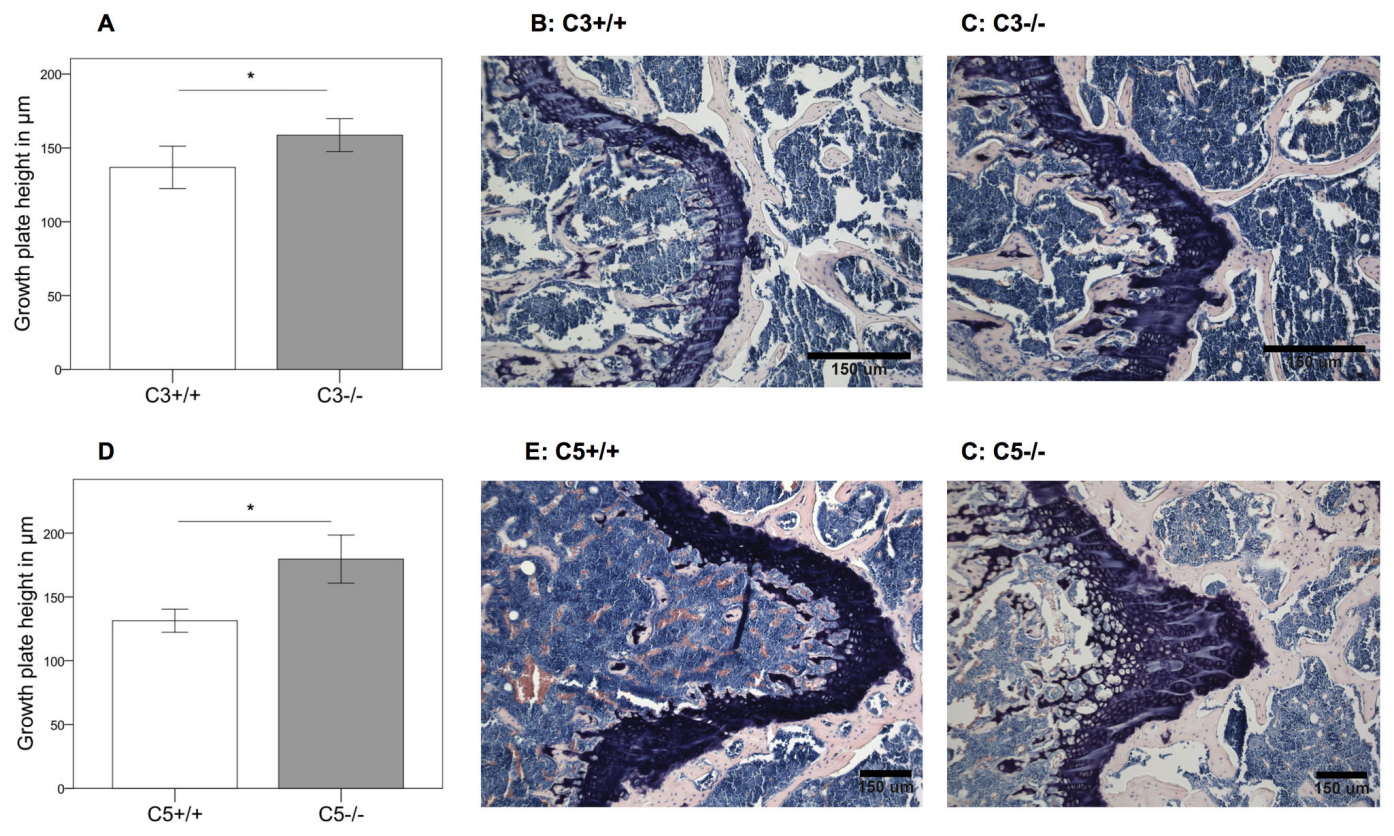
Analyzes of the growth plates. A, D: Growth plate height in complement-deficient and corresponding wildtype mice. Results are displayed as mean ± 2×SEM; * = p≤0.05. B, C, E, F: Histological slides of the growth plates; B: C3^+/+^ mouse; C: C3^-/-^ mouse; E: C5^+/+^ mouse; F: C5^-/-^ mouse.

The epiphyseal growth plate of the proximal femur was higher in C3^-/-^ mice (+14%; p=0.029) (Figure 2 A‑C) and C5^-/-^ animals (+27%; p=0.001) (Figure 2 D‑F). These preliminary results demonstrated that C3- and C5-deficient mice did not display a severe bone phenotype at age 12 weeks but do suggest that complement may play a role in the longitudinal growth of long bones, which should be addressed in further studies.

### Fracture healing in the absence of complement C3

**Figure 3:**
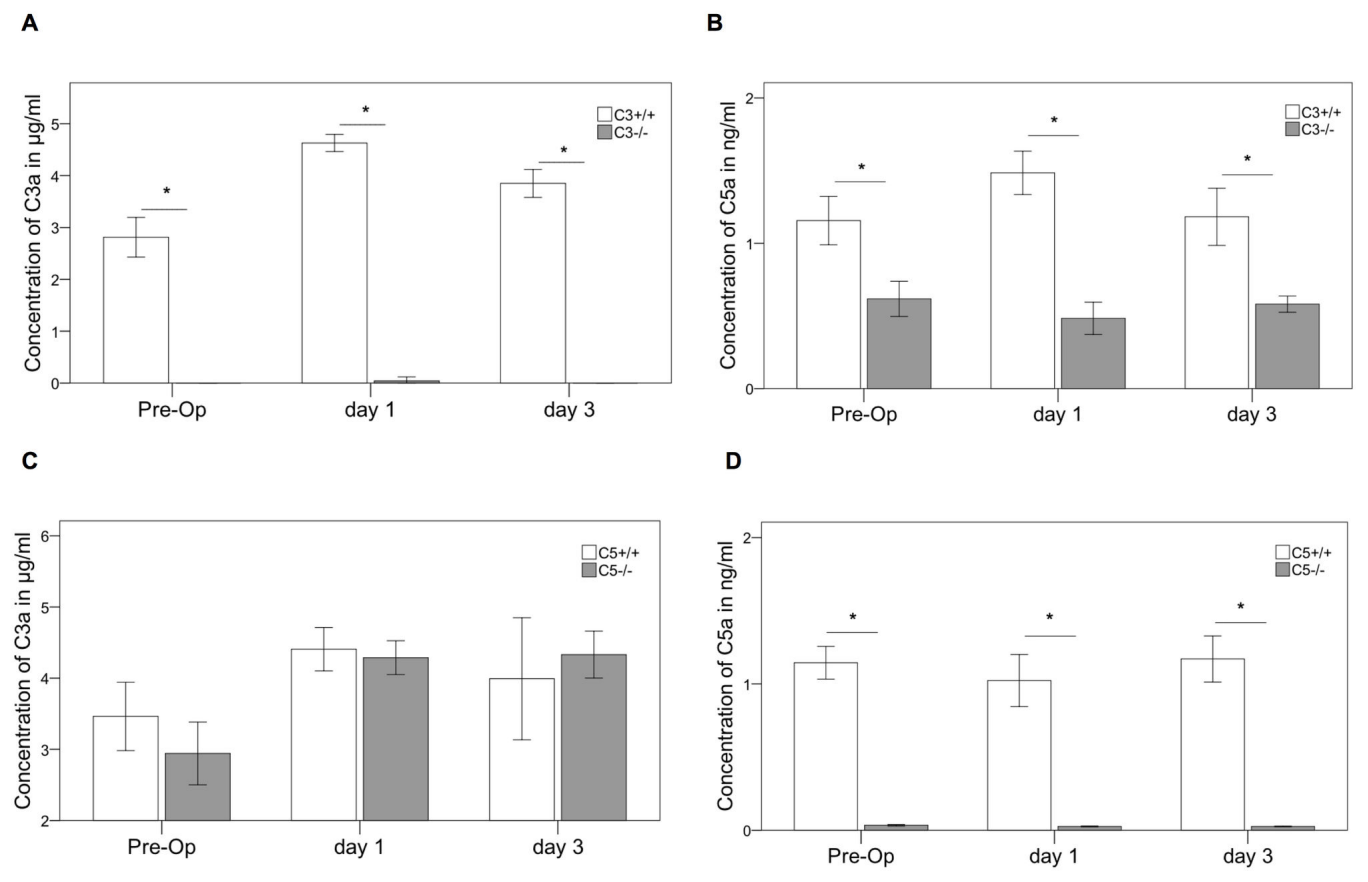
Complement serum analysis. C3a and C5a concentration in the serum preoperatively (Pre-Op), 1 and 3 days after the osteotomy. Results are displayed as mean ± 2×SEM; * = p≤0.05; A: C3a in C3^-/-^; B: C5a in C3^-/-^; C: C3a in C5^-/-^; D: C5a in C5^-/-^.

In wildtype mice systemic C3a slightly increased on day 1 compared to pre-operative values and returned to normal levels on day 3 (Figure 3A and C). As expected, no systemic C3a was measured in C3^-/-^ mice (Figure 3A). The C5a concentration in the serum of C3^-/-^ mice was significantly reduced before, and on day 1 and 3 after fracture (p=0.001) in comparison to the corresponding wildtype (Figure 3B). Notably, C5a could still be measured in the C3^-/-^ mice, confirming that C5 was in addition activated by extrinsic pathways [6,7]. 

**Figure 4:**
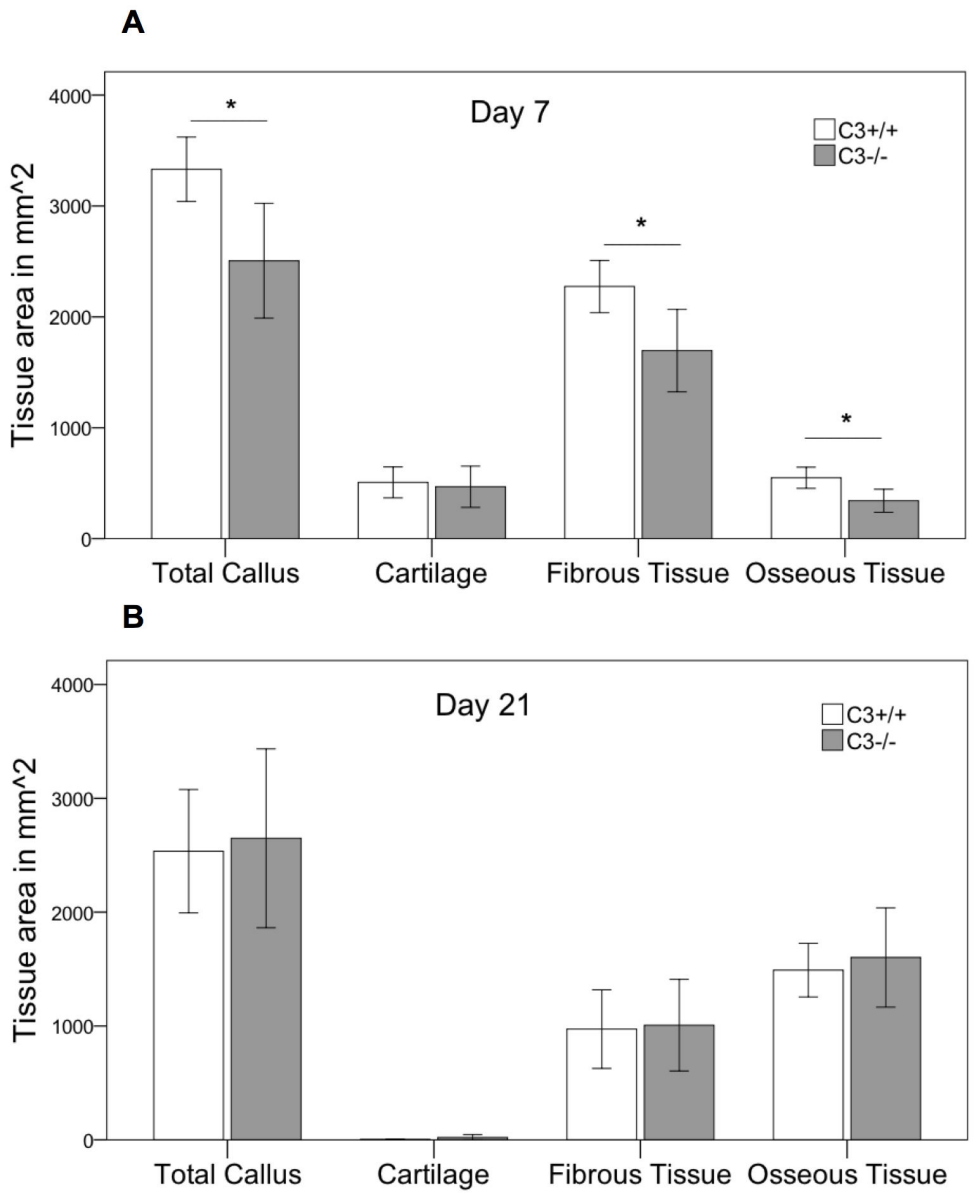
Histological evaluation in C3^-/-^. Histological evaluation of the fracture callus in the absence of C3. Area of the total callus and of cartilage, soft tissue and newly formed bone of the fracture callus of C3^-/-^ and the corresponding wildtype mice. Results are displayed as mean ± 2×SEM; * = p≤0.05; A: day 7 after osteotomy; B: day 21 after osteotomy.

**Figure 5:**
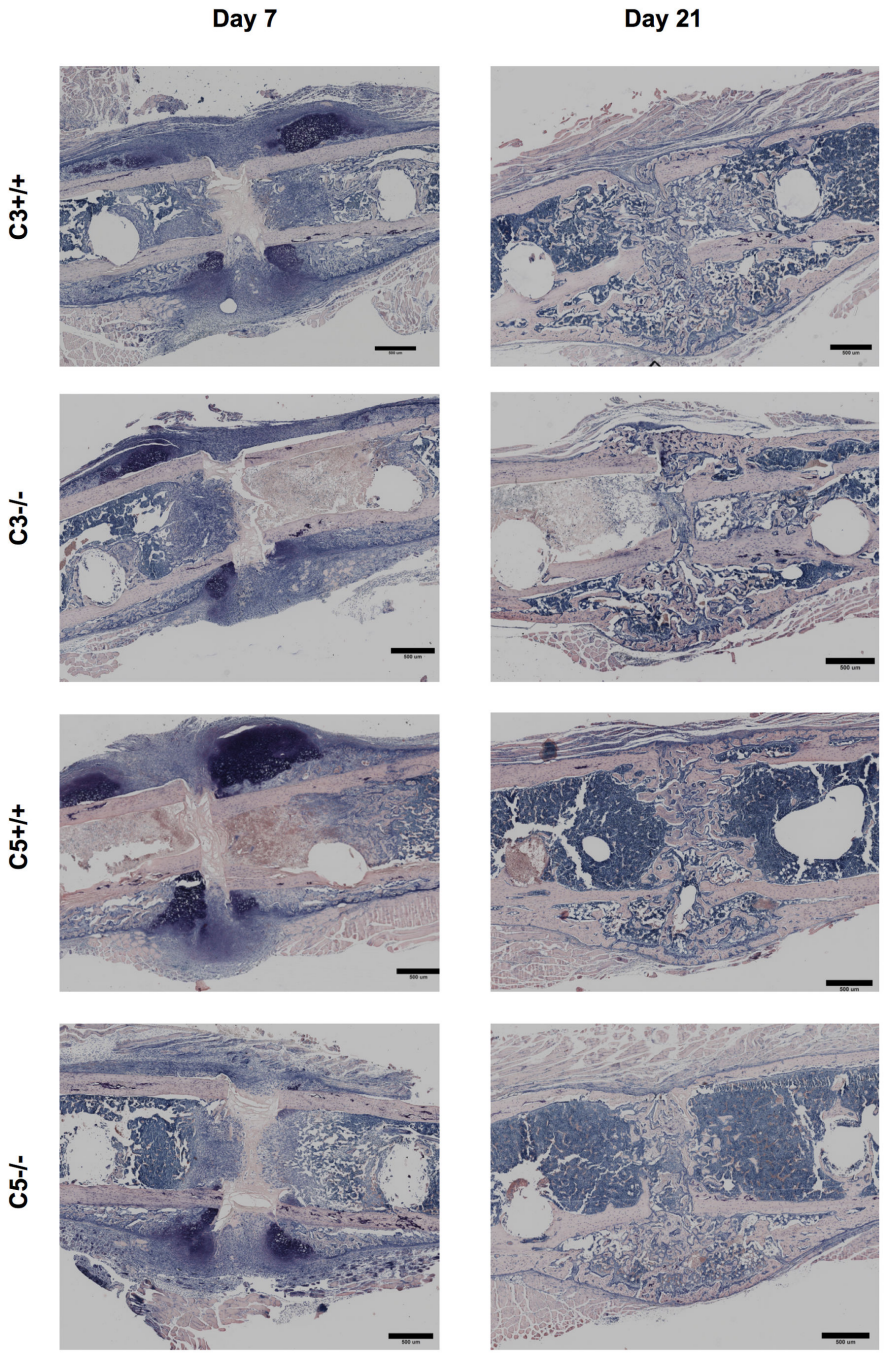
Histology. Histological slides of the fracture callus of complement-deficient and corresponding wildtype mice 7 and 21 days after osteotomy. Giemsa staining. Bar = 200 µm.

**Figure 6:**
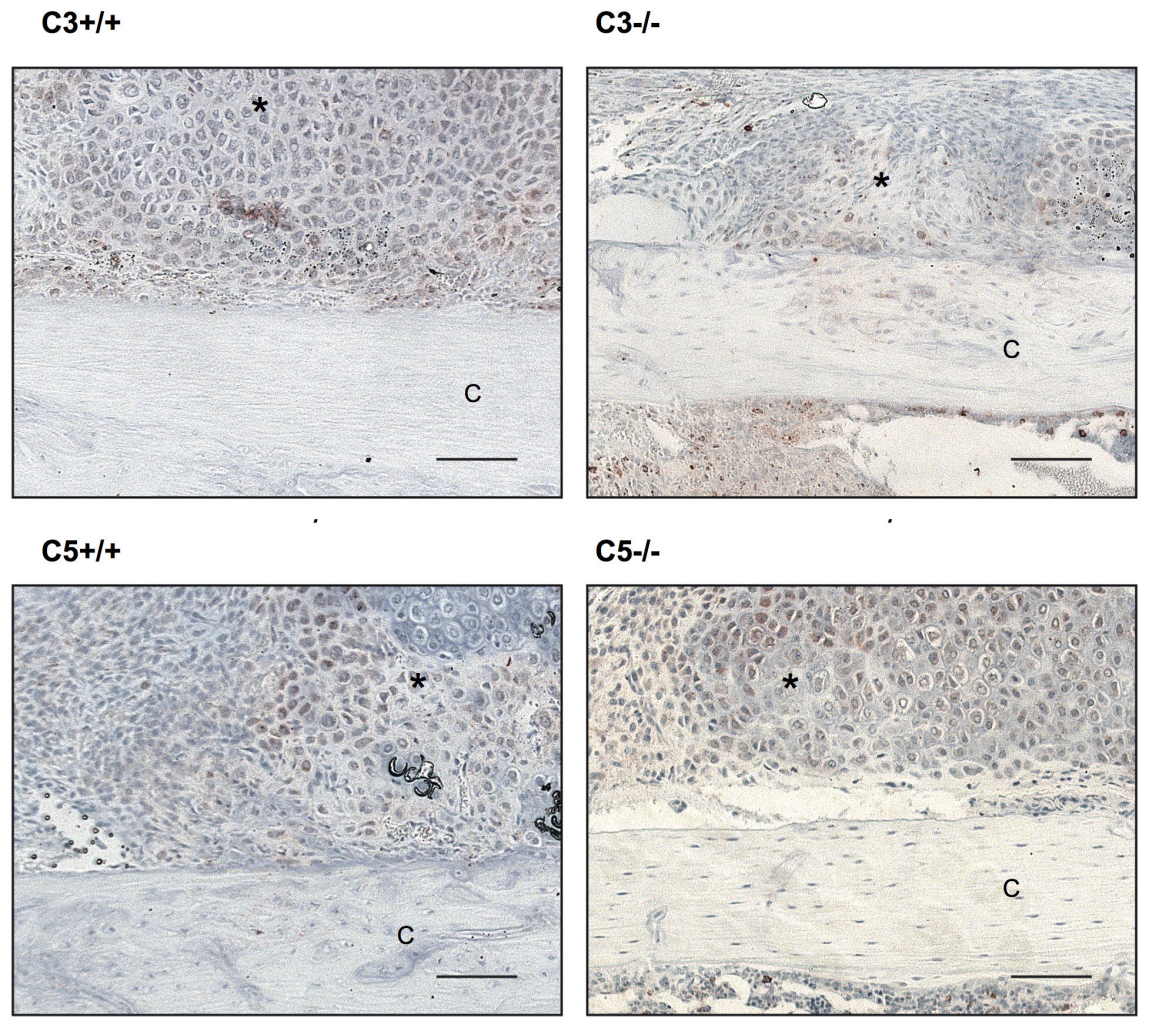
Immunostaining for IL-6. Histological slides from all 4 experimental groups after immunostaining for IL-6. Displayed are sections from the periosteal callus with predominant staining of the hypertrophic chondrocytes. Scale for all slides 100 µm as indicated. C: cortical bone; *: proliferating osteoblasts and hypertrophic chondrocytes.

**Figure 7:**
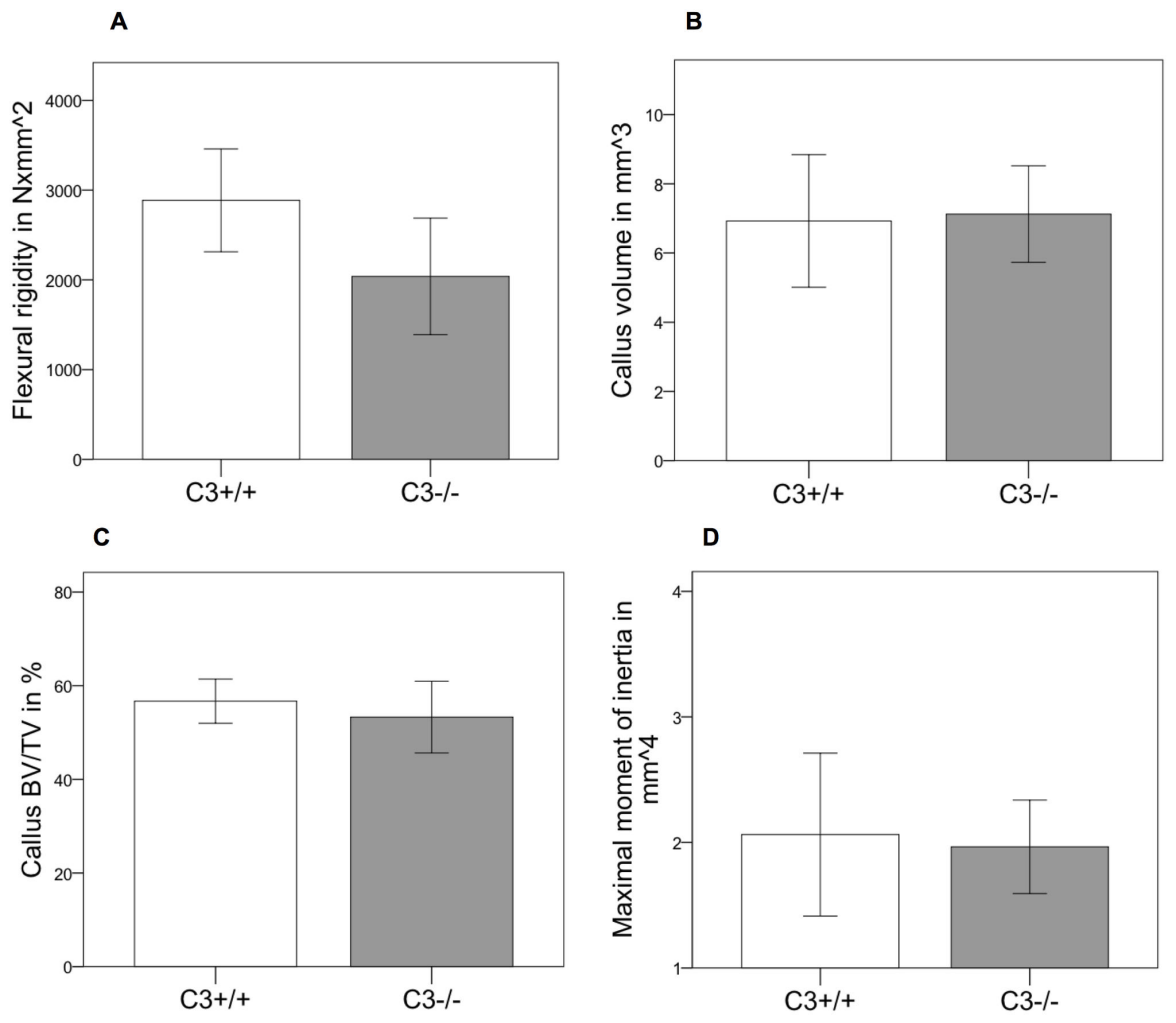
Results fracture healing in C3^-/-^. Biomechanical and µCT analyses of the fracture callus in the absence of C3 at 21 days after the osteotomy. A: Bending stiffness of the fracture callus. B: Total callus volume. C: Bone volume/total volume. D: Maximal moment of inertia. Results are displayed as mean ± 2×SEM; * = p≤0.05.

Histological analysis revealed, that during the early phase of fracture healing (7 days after osteotomy) the overall callus size was significantly reduced by 25% (p=0.02) in the absence of C3 (Figure 4A & Figure 5). The amount of cartilage was not significantly affected at this time point. However, there was considerably less bone (-38%, p=0.01) and less fibrous tissue (-25%, p=0.025) in the callus of C3^-/-^ mice. After 21 days, the time point of cortical bridging, there were no significant differences between both genotypes (Figure 4B & Figure 5). Immunostaining for IL-6 showed no significant differences between C3^-/-^ and their corresponding wildtypes (Figure 6) with mainly proliferating osteoblast precursor cells and chondroblasts positively stained for IL-6. The histological results were confirmed by the biomechanical and µCT evaluation (Figure 7). Taken together, these data indicate that in the early phase of fracture healing, the absence of C3 reduced callus size and bone formation, but not cartilage formation, and that these effects were completely overcome in the late healing phase.

### Fracture healing in the absence of complement C5

As expected, no C5a was measured in the serum of C5^-/-^ mice (Figure 3D). Analysis of C3a showed almost similar values for C5^-/-^ and C5^+/+^ animals with a slight increase of C3a after osteotomy in both animal strains (Figure 3C). 

**Figure 8:**
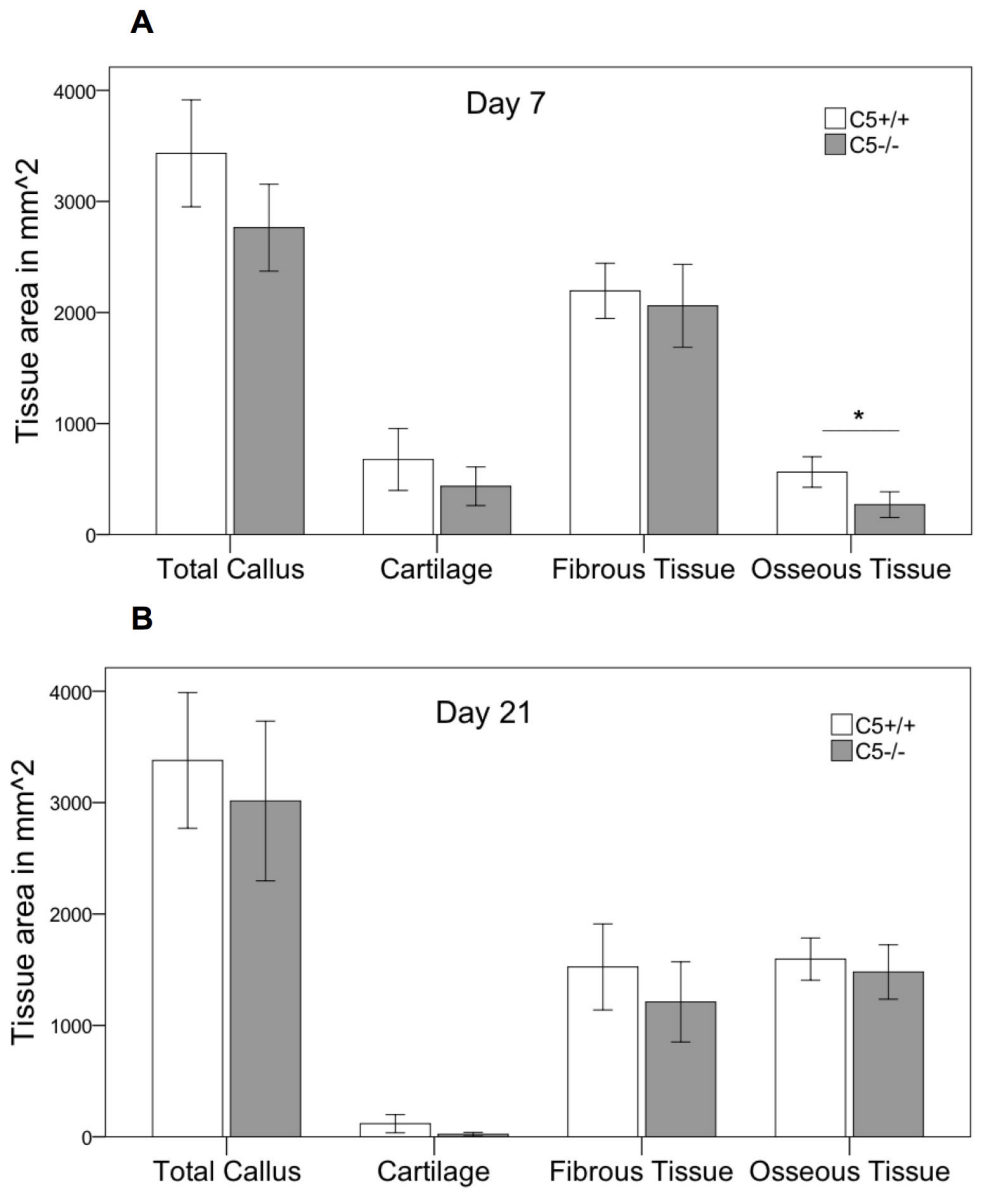
Histological evaluation in C5^-/-^. Histological evaluation of the fracture callus in the absence of C5. Area of the total callus and of cartilage, soft tissue and newly formed bone of the fracture callus of C5^-/-^ and the corresponding wildtype mice. Results are displayed as mean ± 2×SEM; * = p≤0.05; A: day 7 after osteotomy; B: day 21 after osteotomy.

**Figure 9:**
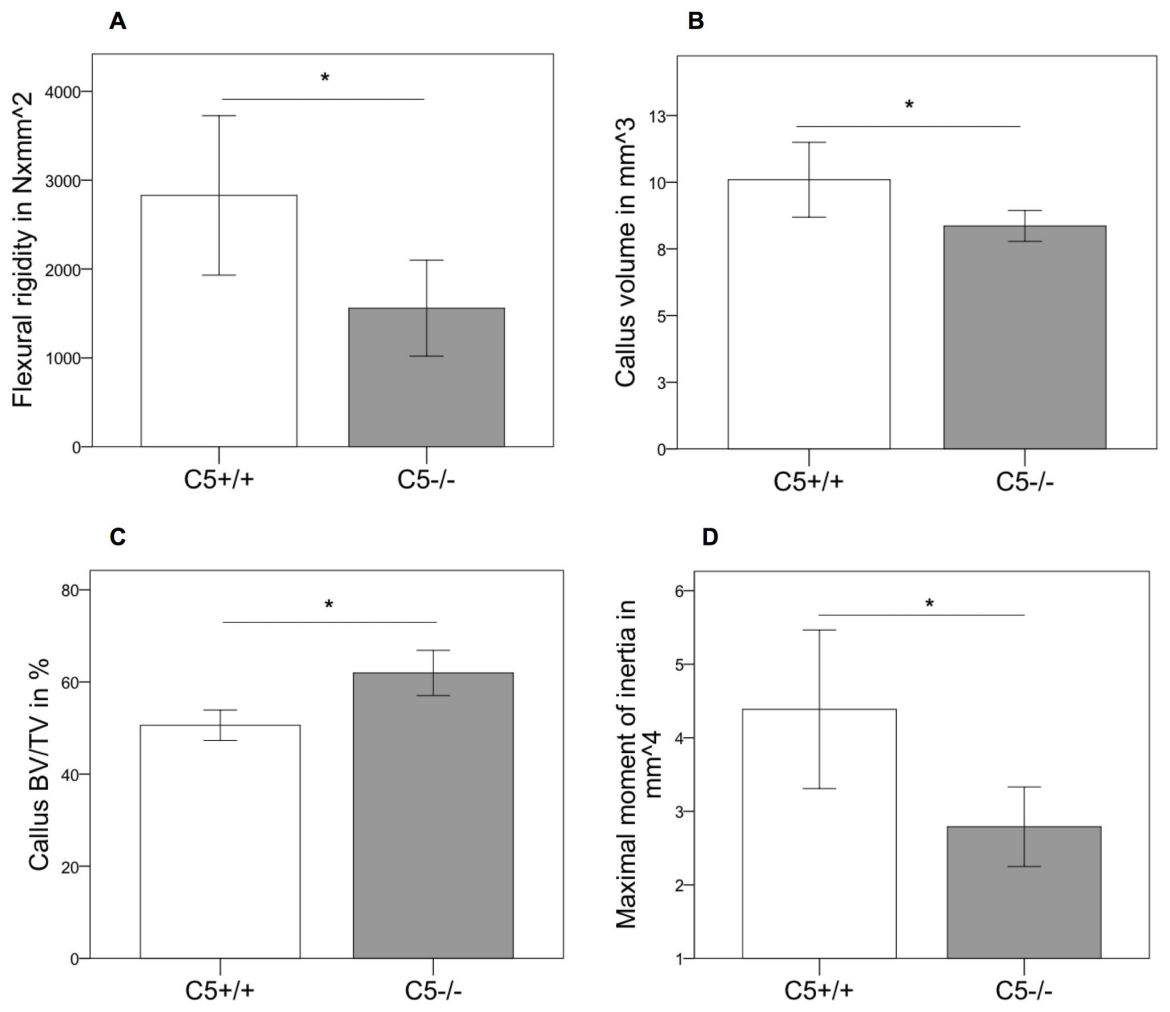
Results fracture healing in C5-/-. Biomechanical and µCT analyses of the fracture callus in the absence of C5 at 21 days after the osteotomy. A: Bending stiffness of the fracture callus. B: Total callus volume. C: Bone volume/total volume. D: Maximal moment of inertia. Results are displayed as mean ± 2×SEM; * = p≤0.05.

Histological analysis revealed some reduction (-20%) in the overall callus size 7 days after osteotomy that failed to reach statistical significance (p=0.052). Corresponding to C3^-/-^ mice, the amount of osseous tissue was significantly diminished by 52% (p=0.009) in the absence of C5 (Figure 8A). After 21 days, no significant differences between C5^-/-^ and wildtype mice were detected in two-dimensional histology (Figure 8B). Corresponding to the results for C3^-/-^ immunostaining for IL-6 showed no significant differences between C5^-/-^ and their corresponding wildtypes with a similar distribution pattern (Figure 6). However, three-dimensional µCT-analysis revealed a significantly diminished overall callus volume (-17%; p=0.039) and moment of inertia (-37%, p=0.042) in the absence of C5 (Figure 9B&D). This corresponds to a significantly reduced bending stiffness in C5-deficient mice (Figure 9A). BV/TV was slightly increased in C5^-/-^ mice, suggesting that the poor mechanical properties of the healed bone mainly resulted from a smaller callus and not from impaired callus quality (Figure 9C).

## Discussion

This study demonstrates that complement plays a regulatory role in fracture healing. In both C3- and C5-deficient mice, bone formation was significantly reduced during the early healing phase. Confirming our hypothesis, fracture healing was severely affected in C5-deficient mice, which also exhibited a smaller callus with decreased mechanical competence in the late healing phase, whereas C3-deficient mice overcame the initial impairment of bone formation and healed normally after 21 days. These results suggest that the terminal complement pathway, which was completely abolished in C5^-/-^ mice, but not in C3^-/-^ mice, may be particularly important for successful fracture healing.

Previous studies reported that complement might play a role in bone development, because several complement components, including C3 and C5, were expressed in a distinct spatial pattern in the growth plate [10]. Whereas C3 was mainly found in the resting zone, C5 was predominantly expressed in the hypertrophic zone of the growth plate where chondrocytes become hypertrophic and are replaced by bone [10]. The authors concluded that complement may contribute to cartilage-bone transformation during endochondral ossification. This was supported by the demonstration that C1s, the first component of the classical pathway of complement activation, which is able to cleave collagen due to its serine protease activity, may play a role in cartilage degradation during ossification [11,13]. Furthermore, it was reported that efficient osteoclast formation is dependent on C3, because C3-deficient bone marrow cells exhibited a lower expression of osteoclast stimulation factors (e.g. IL-6) and generated significantly fewer osteoclasts than wild-type bone marrow cells [28]. Therefore, we analyzed the bone phenotype of C3- and C5-deficient mice. The skeletons of both genotypes exhibited normal size and shape at age 12 weeks. Whereas trabecular bone of the vertebrae was not influenced by the absence of C3 or C5, there were indications for an influence of C3 and C5 on cortical bone. The stiffness of the femoral shaft was slightly decreased in the absence of C3 or C5. The width of the growth plate was enhanced, particularly in C5^-/-^ mice, indicating that endochondral ossification could be delayed in the absence of C3 and C5. These data confirm previously published results [11,13], and additionally suggest that not only early complement components, such as C1s [11,13], but also the terminal pathway may be important in endochondral ossification. A limitation of this study is that only the phenotype of 12-week old mice, which were used for the fracture healing experiments, were investigated. Additional studies need to be conducted on both younger and older mice to further analyze the role of complement in bone development.

Our previous data suggest that complement may also play a role in fracture healing [19,29]. We demonstrated that the key complement receptor C5aR was abundantly expressed in the fracture callus of rats not only by immune cells during the early inflammatory phase but also by osteoblasts, chondroblasts and osteoclasts throughout the entire healing period [17]. The expression patterns of C5aR and C3aR were similar (unpublished results of our group). The ligands of these receptors (complement anaphylatoxins C3a and C5a) may also be present during all stages of fracture healing. Immediately after injury, complement is activated locally as an early defense mechanism against endogenous or exogenous pathogens, such as destroyed tissue and microorganisms, triggering the local immune response [4]. After the initial inflammatory response, C5a may also be produced by bone cells at the fracture site. It has been shown that osteoblasts secrete C3 and C5 [19], and that osteoclasts are able to effectively cleave C5 to its active form C5a [19]. The results of the present study on complement-deficient mice confirmed the involvement of complement in fracture healing. During the early phase of fracture healing, a smaller callus and slightly reduced bone formation in the absence of C3 and C5 was demonstrated, whereas cartilage formation was not affected. C5a is a strong chemoattractant for immune cells [30] and induces the migration of mesenchymal stem cells and osteoblasts [17,22]. The smaller callus in complement-deficient mice may result from a decreased inflammatory reaction and a reduced recruitment of osteoblast precursor cells in the early fracture hematoma. During the early phase, bone is mainly formed by intramembranous ossification near the periosteum at some distance from the fracture gap [31]. The reduced amount of bone in C3- and C5-deficient mice observed after 7 days may indicate an involvement of complement in direct bone formation, possibly by influencing the expression of regulatory cytokines in a paracrine or autocrine fashion. We and other groups have shown that C5a not only induces osteoblast migration but also modifies their release of interleukin-6 (IL-6) [19,21]. IL-6 is regarded as a key cytokine for the initiation of the repair process during fracture healing [32]. Its significance arises from studies in IL-6-deficient mice, which exhibited reduced callus mineralization, particularly during the early phases of fracture healing [33]. Our immune histological results revealed no differences in IL-6 expression in C3^-/-^ and C5^-/-^mice compared to the corresponding wildtypes. Mainly proliferating osteoblast precursor cells and chondroblasts were positively stained for IL-6, which is in accordance with the literature [34]. This indicates that IL-6 may not be involved in the pathomechanisms of disturbed fracture healing in complement deficient mice. 

Whereas in C3^-/-^ mice the reduced bone formation observed during the early stage of fracture healing was overcome after 21 days, bone repair was considerably impaired in the absence of C5. This was confirmed by a significantly reduced mechanical competence of the fractured bone and a smaller callus. The relative amount of bone was not reduced, rather even slightly increased, suggesting that the cartilage was successfully transformed to bone during endochondral ossification. These results confirm the initial hypothesis of this study, with an expected stronger effect on bone healing in C5-deficient mice. In C3^-/-^ mice, C5 can still be activated by extrinsic pathways. It was demonstrated that C5 can be effectively cleaved to C5a by thrombin [6] and by membrane-bound serine proteases of macrophages and neutrophils independently of C3-activation [7]. These extrinsic activation pathways may in particular play a role in complement activation after injury [6]. This was confirmed by serum measurements of C5a, where relevant amounts of C5a were found in C3^-/-^ mice. The only plausible source for systemic C5a may be C5 cleavage via thrombin generated at the fracture site.

However, the present C5a levels measured in serum may be overestimated based on the coagulation-induced activation of complement as recently shown [6,35] even though the samples were strictly kept on ice.

The presented results suggest that regular fracture healing requires an unimpaired complement system with functional C5a. This hypothesis is supported by the fact that C5 was demonstrated to be important for limb regeneration and displayed robust expression in wound epithelium [36]. However, the complement system is not only known to exert beneficial effects. In the experimental setting of severe blunt chest trauma in combination with a femur fracture, complement activation induced detrimental effects on fracture healing, which could be reversed by systemic blockade of the C5a-C5aR interaction [37]. These results are in accordance with other studies demonstrating a negative role for systemic complement activation during massive inflammatory reactions, including in sepsis and multiple-organ dysfunction [38-40]. In summary, it appears that a balanced activation of complement is required to enable bone regeneration and that an overstimulation of complement provokes deleterious effects.

## Conclusion

Taken together, complement was shown to be crucially involved in fracture healing. Because fracture healing was severely affected in the absence of C5, the terminal complement pathway appears to play an important role. Changes in C3^-/-^ mice were less profound, probably because of a cross activation of complement by the coagulation cascade. Future studies should evaluate the involvement of different complement proteins in bone development and during the time-course of fracture healing 
